# A metagene based similarity network fusion approach for multi-omics data integration identified novel subtypes in renal cell carcinoma

**DOI:** 10.1093/bib/bbae606

**Published:** 2024-11-19

**Authors:** Congcong Jia, Tong Wang, Dingtong Cui, Yaxin Tian, Gaiqin Liu, Zhaoyang Xu, Yanhong Luo, Ruiling Fang, Hongmei Yu, Yanbo Zhang, Yuehua Cui, Hongyan Cao

**Affiliations:** Department of Health Statistics, Shanxi Provincial Key Laboratory of Major Diseases Risk Assessment, School of Public Health, Shanxi Medical University, Taiyuan, Shanxi, 030001, PR, China; MOE Key Laboratory of Coal Environmental Pathogenicity and Prevention, Shanxi Medical University, Taiyuan, Shanxi, 030001, PR, China; Department of Health Statistics, Shanxi Provincial Key Laboratory of Major Diseases Risk Assessment, School of Public Health, Shanxi Medical University, Taiyuan, Shanxi, 030001, PR, China; Academy of Medical Sciences, Shanxi Medical University, Taiyuan, Shanxi, 030001, PR, China; Department of Health Statistics, Shanxi Provincial Key Laboratory of Major Diseases Risk Assessment, School of Public Health, Shanxi Medical University, Taiyuan, Shanxi, 030001, PR, China; MOE Key Laboratory of Coal Environmental Pathogenicity and Prevention, Shanxi Medical University, Taiyuan, Shanxi, 030001, PR, China; Department of Health Statistics, Shanxi Provincial Key Laboratory of Major Diseases Risk Assessment, School of Public Health, Shanxi Medical University, Taiyuan, Shanxi, 030001, PR, China; Academy of Medical Sciences, Shanxi Medical University, Taiyuan, Shanxi, 030001, PR, China; Department of Health Statistics, Shanxi Provincial Key Laboratory of Major Diseases Risk Assessment, School of Public Health, Shanxi Medical University, Taiyuan, Shanxi, 030001, PR, China; MOE Key Laboratory of Coal Environmental Pathogenicity and Prevention, Shanxi Medical University, Taiyuan, Shanxi, 030001, PR, China; Department of Health Statistics, Shanxi Provincial Key Laboratory of Major Diseases Risk Assessment, School of Public Health, Shanxi Medical University, Taiyuan, Shanxi, 030001, PR, China; Academy of Medical Sciences, Shanxi Medical University, Taiyuan, Shanxi, 030001, PR, China; Department of Health Statistics, Shanxi Provincial Key Laboratory of Major Diseases Risk Assessment, School of Public Health, Shanxi Medical University, Taiyuan, Shanxi, 030001, PR, China; MOE Key Laboratory of Coal Environmental Pathogenicity and Prevention, Shanxi Medical University, Taiyuan, Shanxi, 030001, PR, China; Department of Health Statistics, Shanxi Provincial Key Laboratory of Major Diseases Risk Assessment, School of Public Health, Shanxi Medical University, Taiyuan, Shanxi, 030001, PR, China; MOE Key Laboratory of Coal Environmental Pathogenicity and Prevention, Shanxi Medical University, Taiyuan, Shanxi, 030001, PR, China; Department of Health Statistics, Shanxi Provincial Key Laboratory of Major Diseases Risk Assessment, School of Public Health, Shanxi Medical University, Taiyuan, Shanxi, 030001, PR, China; MOE Key Laboratory of Coal Environmental Pathogenicity and Prevention, Shanxi Medical University, Taiyuan, Shanxi, 030001, PR, China; Department of Health Statistics, Shanxi Provincial Key Laboratory of Major Diseases Risk Assessment, School of Public Health, Shanxi Medical University, Taiyuan, Shanxi, 030001, PR, China; MOE Key Laboratory of Coal Environmental Pathogenicity and Prevention, Shanxi Medical University, Taiyuan, Shanxi, 030001, PR, China; Department of Statistics and Probability, Michigan State University, East Lansing, MI, 48824, United States; Department of Health Statistics, Shanxi Provincial Key Laboratory of Major Diseases Risk Assessment, School of Public Health, Shanxi Medical University, Taiyuan, Shanxi, 030001, PR, China; MOE Key Laboratory of Coal Environmental Pathogenicity and Prevention, Shanxi Medical University, Taiyuan, Shanxi, 030001, PR, China

**Keywords:** Meta-SNF, RCC, multi-omics data integration, subtypes identification, non-negative matrix factorization

## Abstract

Renal cell carcinoma (RCC) ranks among the most prevalent cancers worldwide, with both incidence and mortality rates increasing annually. The heterogeneity among RCC patients presents considerable challenges for developing universally effective treatment strategies, emphasizing the necessity of in-depth research into RCC’s molecular mechanisms, understanding the variations among RCC patients and further identifying distinct molecular subtypes for precise treatment. We proposed a metagene-based similarity network fusion (Meta-SNF) method for RCC subtype identification with multi-omics data, using a non-negative matrix factorization technique to capture alternative structures inherent in the dataset as metagenes. These latent metagenes were then integrated to construct a fused network under the Similarity Network Fusion (SNF) framework for more precise subtyping. We conducted simulation studies and analyzed real-world data from two RCC datasets, namely kidney renal clear cell carcinoma (KIRC) and kidney renal papillary cell carcinoma (KIRP) to demonstrate the utility of Meta-SNF. The simulation studies indicated that Meta-SNF achieved higher accuracy in subtype identification compared with the original SNF and other state-of-the-art methods. In analyses of real data, Meta-SNF produced more distinct and well-separated clusters, classifying both KIRC and KIRP into four subtypes with significant differences in survival outcomes. Subsequently, we performed comprehensive bioinformatics analyses focused on subtypes with poor prognoses in KIRC and KIRP and identified several potential biomarkers. Meta-SNF offers a novel strategy for subtype identification using multi-omics data, and its application to RCC datasets has yielded diverse biological insights which are highly valuable for informing clinical decision-making processes in the treatment of RCC.

## Introduction

Renal cell carcinoma (RCC), commonly derived from pathological changes in renal tubular epithelial cells [1], is among the most prevalent types of cancer worldwide and has a higher incidence rate compared to other urologic cancers [2–4]. Despite advancements in RCC diagnosis and treatment over decades, the incidence and mortality rates of RCC continue to rise each year [5]. Currently, Stage I to III of RCC can be effectively treated with surgical resection, but the recurrence rate can be as high as one-third [6]. In cases of advanced or metastatic RCC, targeted therapy and immunotherapy constitute the primary treatment approaches. However, it is noteworthy that these methods are effective only in a subset of patients and may lead to the development of drug resistance [7]. Heterogeneity among patients makes it challenging to replicate individual treatment plans across the population, and the evolution of tumor cells undermines the effectiveness of the current treatment system [8]. Thus, it is still crucial to conduct extensive research into the molecular mechanisms of RCC and capture the heterogeneity between RCC patients to identify molecular subtypes for developing comprehensive treatment strategies.

Tumor development is widely recognized as a complex process influenced by factors such as genetic mutations, abnormal gene expression, and heredity. High-throughput ‘multi-omics’ technologies provide a robust foundation for studying these influences [9]. Recently, integrating multi-omics data has become key in understanding tumor pathogenesis and molecular subtyping [10], revealing disease mechanisms from multiple perspectives [11]. Multi-omics studies present data that vary in type, scale, and distribution, often encompassing thousands of variables yet limited to a small number of samples. These biological datasets are inherently complex, characterized by a plethora of noisy features, and susceptible to potential measurement errors or biological deviations [12]. To tackle these challenges, numerous computational methods and statistical strategies have been developed, broadly categorized into four classes [12–14]. One class is called ‘early integration’ which involves concatenating all omics data matrices but struggles with high-dimensional, noisy data, and often overlooks the heterogeneity of multi-omics datasets [15, 16]. Another class is called ‘late integration’, exemplified by strategies like COCA [17], which first applies clustering algorithms to individual omics data types and then uses ensemble methods on these results for clustering. Its drawback lies in the potential loss of complementary information across different omics data, which may hinder the discovery of underlying biological mechanisms of diseases.

The third integration strategy relies on similarity-based methods which transform input data into a similarity matrix, addressing early integration’s limitations and easing subsequent integration. A popular method in this category is the Similarity Network Fusion (SNF) [18] which constructs sample similarity networks for each omics type using a scaled exponential kernel function, then fuses them into a single network through a message passing algorithm [19], effectively capturing similarity among samples. However, the inevitable technical error and biological noise may introduce potential spurious associations between patients [20, 21]. The fourth category is known as ‘intermediate integration’ which typically creates new representations for multi-omics datasets, and operates on the premise that different datasets share a common latent space [12–14]. Further analyses, such as extensions of the non-negative matrix factorization (NMF) [22] methods (e.g., intNMF [23]), can then be performed. NMF, a potent matrix factorization technique, excels in uncovering alternate structures within datasets (e.g., metagenes), and has been widely applied in various fields including image processing [24], text-mining [25], and machine learning [26]. Its broad applicability extends to the exploration of cancer molecular mechanisms, where it plays a crucial role in facilitating subtypes identification. Moreover, metagenes extracted by the NMF algorithm reduce dimensionality and noise in molecular data while capturing biological relevance, strongly supporting the exploration of potential biological mechanisms.

This underscores the complementarity between the two integration strategies: NMF and SNF, each offering unique advantages from different perspectives. To capitalize on the strengths of these strategies for more effective subtyping, we integrated the two frameworks and proposed a novel multi-omics integration method called Metagene-based Similarity Network Fusion (Meta-SNF). It involves using the NMF technique to identify latent structures as metagenes within each data type, thereby reducing the noise while enhancing data representativeness. These metagenes are then integrated to construct a fused network within the SNF framework. We conducted simulation studies to validate our hypothesis by comparing Meta-SNF’s performance with the original SNF method and their counterparts, specifically intNMF [23] and ConsensusClusterPlus [27]. To further evaluate the capability of Meta-SNF with respect to real RCC data, we further applied it to molecular subtyping of multi-omics data in two types of RCC from The Cancer Genome Atlas Program (TCGA) [28]: kidney renal clear cell carcinoma (KIRC) and kidney renal papillary cell carcinoma (KIRP), which together represent ~ 95% of all RCCs [8]. For the subtypes identified by Meta-SNF, extensive biological analyses were performed to identify potential molecular biomarkers that could influence RCC progression. Our findings confirms that Meta-SNF has better clustering accuracy compared to the original SNF algorithm and is capable of identifying RCC subtypes with significant survival differences, offering a new integration strategy and perspective for research involving multi-omics data integration for RCC subtyping.

## Methods

### Meta-SNF

**Figure 1:**
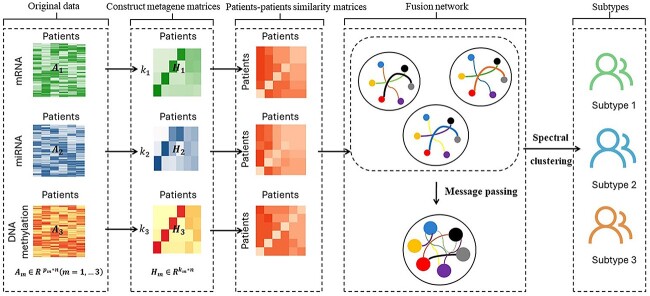
The workflow of Meta-SNF. Flowchart of Meta-SNF illustrated with three omics data types of patients, namely miRNA, mRNA, and DNA methylation, which firstly were extracted their own underlying structures by NMF and defined as metagenes matrices respectively for each omics. Then patients-patients similarity matrices for each data type are constructed based on these metagenes matrices, which were further updated iteratively through message passing, making them more similar with each other and resulting in final fusion network at convergence. Ultimately, patients were classified into different subtypes with spectral clustering.

Meta-SNF incorporates the NMF algorithm into the process of SNF, which first uses NMF to extract the underlying structures, defined as metagenes and separately for each data type, and then integrates different metagenes into a fused network under the SNF framework for further subtyping. Each metagene is a latent variable representing a low dimensional projection of the original omics data types. Metagenes have lower noise levels compared to the original data. Thus, the fused network constructed using the metagenes can better capture the relationships among samples, leading to a more reliable molecular subtyping outcome. The implementation process of Meta-SNF is illustrated in Fig. 1, with specific steps outlined below:

*Construct metagenes using NMF*

Let ${A}_m\in{R}^{p_m\times n}$ denote the *m*th ($m=1,\cdots, M$) omics data matrix with ${p}_m$ features and $n$ samples, where ${\left({A}_m\right)}_{ij}\ge 0$ ($i=1,2,\cdots, {p}_m,j=1,2,\cdots, n$). We apply the NMF algorithm [22, 29] to decompose the matrix ${A}_m$. Through iterative parameter tuning, our goal is to find the optimal non-negative matrix ${W}_m\in{R}^{p_m\times{k}_m}$(where ${w}_{il}\ge 0,l=1,2,\cdots, {k}_m$) and matrix ${H}_m\in{R}^{k_m\times n}$ (where ${h}_{lj}\ge 0$) such that ${A}_m\approx{W}_m{H}_m$. Here, the entry ${w}_{il}$ in ${W}_m$ is the coefficient of gene $i$ in metagene $l$. ${H}_m$ is the metagene matrix for the $m$th data type, in which ${h}_{lj}$ represents the expression level of metagene $l$ in sample $j$.

Choosing the appropriate rank ${k}_m$ is crucial to the stability of cluster results, which also defines the number of metagenes. Here, we adopt the cophenetic correlation coefficient idea [22]. The cophenetic correlation coefficient reflects the stability of the clusters obtained from NMF, calculated as the Pearson correlation between sample distances induced by the consensus matrix and the cophenetic distances obtained through hierarchical clustering. Here, we select the first of two consecutive $k$ values that corresponds to the largest difference in cophenetic correlation coefficients as the final rank ${k}_m$. The construction of matrices ${W}_m$ and ${H}_m$ is determined by the rank ${k}_m$ and dimensions of ${A}_m$. They are randomly initialized but iteratively updated to minimize the Generalized Kullback–Leibler Divergence (GKLD) [30] that quantifies the difference between the matrix ${A}_m$ and the product of ${W}_m\ \textrm{and}\ {H}_m$ as given in Algorithm 1. Algorithm 1 summarizes the procedure of NMF.

**Algorithm 1:** NMF (Non-negative matrix factorization)

**Input:** non-negative matrix ${A}_m$; initialized matrices ${W}_m$ and ${H}_m$; rank ${k}_m$. **Output:** optimized matrices ${W}_m$ and ${H}_m$ such that ${A}_m\approx{W}_m{H}_m$. **Details:** 1. Identify the rank ${k}_m$ based on the cophenetic correlation coefficient and generate the initialized matrices ${W}_m$ and ${H}_m$. 2. Set the number of runs to perform NMF (default 30 times); minimize the Generalized Kullback–Leibler Divergence which is the divergence between matrices ${A}_m$ and ${W}_m{H}_m$, as follows: $\kern1.5pc\underset{W,H\ge 0}{\mathit{\min}} KL\left({A}_m;{W}_m{H}_m\right)=\sum\limits_{i,j}\left({\left({A}_m\right)}_{ij}\log \frac{{\left({A}_m\right)}_{ij}}{{\left({W}_m{H}_m\right)}_{ij}}-{\left({A}_m\right)}_{ij}+{\left({W}_m{H}_m\right)}_{ij}\right).$ 3. Update matrices ${W}_m$ and ${H}_m$ at each iteration by: $\kern5pc{\left({W}_m\right)}_{ia}\leftarrow{\left({W}_m\right)}_{ia}\frac{\sum_j\frac{{\left({A}_m\right)}_{ij}}{{\left({W}_m{H}_m\right)}_{ij}}}{\sum_j{\left({W}_m\right)}_{ja}}{\left({H}_m\right)}_{aj},$ and ${\left({H}_m\right)}_{aj}\leftarrow{\left({H}_m\right)}_{aj}\sum_i{\left({W}_m\right)}_{ia}\frac{{\left({A}_m\right)}_{ij}}{{\left({W}_m{H}_m\right)}_{ij}}.$ 4. Choose the final matrix ${H}_m$ as the metagene matrix after the completion of iterations.

*Integrate metagene matrices using SNF*

SNF integrates multi-omics data by fusing similarity networks derived from different omics data types. Initially, it constructs sample similarity networks for each data type using a similarity kernel function. Subsequently, it utilizes a non-linear approach based on the message-passing theory to integrate these similarity networks into a fused similarity network that captures local structures across diverse data types and harnesses the complementary information within networks [18]. For any two samples $a$ and $b$ in the metagene matrix, their similarity $S\left(a,b\right)$ can be calculated by the similarity kernel function, $S\left(a,b\right)=\mathit{\exp}\left(-\frac{d^2\left(a,b\right)}{\mu{\varepsilon}_{a,b}}\right)$, where $d\left(a,b\right)$ denotes the Euclidean distance between samples $a$ and $b$, $\mu$ is a hyperparameter that is empirically set as 0.5, ${\varepsilon}_{a,b}$ is used to remove the effect due to scaling and can be computed by ${\varepsilon}_{a,b}=\frac{mean\left(d\left(a,{N}_a\right)\right)+ mean\left(d\left(b,{N}_b\right)\right)+d\left(a,b\right)}{3}$ where ${N}_a$ and ${N}_b$ respectively represent the neighbor sample set for $a$ and $b$. Using the metagene matrix ${H}_m$, we construct a sample-sample similarity matrix ${S}_m$. We then define two distinct similarity networks from ${S}_m$: a normalized network ${P}_m$ that contains the global information and a similarity network ${D}_m$ that measures the local affinity. We then iteratively apply the message-passing algorithm to update similarity networks iteratively to achieve network fusion. The procedure is summarized in Algorithm 2.

**Algorithm 2:** Steps of similarity network fusion

**Input**: metagene matrix ${H}_m$ (*m* = 1, …, *M*) **Output**: Fused network$G$ **Steps:** 1. Construct the sample similarity matrix ${S}_m$ via a similarity kernel function. 2. Define normalized network ${P}_m$ and local affinity network ${D}_m$ as follows: ${P}_m\left(a,b\right)=\left\{\begin{array}{@{}l}\frac{S_m\left(a,b\right)}{2{\sum}_{f\ne a}{S}_m\left(a,f\right)},b\ne a\\{}1/2,\kern5.8em b=a\end{array}\kern-4pt\right.$, and ${D}_m\left(a,b\right)=\left\{\begin{array}{@{}l}\frac{S_m(\left(a,b\right)}{\sum_{f\in{N}_a}{S}_m(\left(a,f\right)},\ b\in{N}_a\\{}0, \kern7.8em otherwise.\end{array}\right.$ 3. Update the above similarity networks and fuse them to get the final fusednetwork *G* using a message-passing algorithm as: $\kern4pc G=\frac{\sum_m^M\left({D}^m\times \left\{\frac{\sum_{f\ne m}{P}^{(f)}}{M-1}\right\}\times{\left({D}^{(m)}\right)}^T\right)}{M},m=1,2,\cdots M$

The fused network $G$ can robustly capture similarity information between samples based on multiple metagene matrices obtained from different omics data types. We then apply the spectral clustering method [31] on *G* to obtain subtype clusters. Spectral clustering has fine adaptability to diverse data distributions and yields robust clustering results [32].

### Simulation study

To assess the robustness and performance of the Meta-SNF method in subtype identification based on multi-omics data, we conducted simulation studies to compare it with the original SNF and other multi-omics clustering algorithms, namely intNMF [23] and ConsensusClusterPlus [27].

#### Simulation settings

Following established procedures outlined in the literature [33–35], we generated simulation data with four subtype groups across three omics data types. Each omics dataset contains 200 samples with 1000 features. Within each omics data type, three sample clusters were assumed. Together, the three omics data defined four subtypes with each containing 50 samples. The overall sample label can only be obtained by integrating all three omics datasets. We denoted the three omics data matrices as ${X}^1$, ${X}^2$, and ${X}^3$. To construct the clustering structure of different omics data types, we set ${x}_{pq}^s={mean}^s+{\varepsilon}_{pq}\ \left(p=1,2,\cdots, 200,q=1,2,\cdots, 1000\right)$, where ${mean}^s$ ($s\in \left\{1,2,3\right\}$) represents the mean expression level of features in the *s*th omics data type and ${\varepsilon}_{pq}\sim N\left(0,{\sigma}^2\right)$. Specifically, we set ${mean}^1=\left\{1,0,3\right\}$ for samples {1-50}, {51-150}, and {151-200} in ${X}^1$, ${mean}^2=\left\{0,2,3\right\}$ for samples {1-50}∪{10-150}, {51-100} and {151-200} in ${X}^2$, and ${mean}^3=\left\{2,1,3\right\}$ for samples {1-100}, {101-150}, and {151-200} in ${X}^3$. To assess the robustness of the methods under different sampling variations (i.e., noise levels), we considered three noise levels. In SimData1, SimData2 and SimData3, we set ${\sigma}^2=6,9,\textrm{and}\ 12$, respectively representing low, medium, and high noise level. We also varied the proportion of features that can distinguish the subtypes and they were set as 10%, 15%, and 20%, to investigate the impact of signal strength on the subtyping performance among different methods. We repeated 1000 simulation runs for each scenario.

#### Simulation result evaluation

We used the normalized mutual information (NMI) [36] as the criterion to evaluate the clustering performances of different methods. NMI quantifies the similarity and the level of agreement between the true and estimated clusters, ranging from 0 to 1; a higher NMI indicates better performance of a clustering method. It is defined as:


$$ NMI=\frac{I\left(U,V\right)}{\sqrt{H(U)H(V)}} $$

where *U* and *V* respectively represent the true and the estimated clusters, $I\left(U,V\right)$ is the mutual information between them, and $H\left(\bullet \right)$ is an entropy function.

### Real data analysis

#### RCC datasets and data preprocessing

We applied our method to two renal cell carcinoma multi-omics datasets, KIRC and KIRP, downloaded from the TCGA website using the R package TCGAbiolinks [37]. We considered three omics data types of each cancer, namely miRNA expression, mRNA expression, and promoter CpG methylation. For promoter CpG methylation, we utilized TCGA methylation profile files. Promoter CpGs were defined as methylations located specifically within the promoter region, up to 2 kb from the transcription start site [38], with those on the sex chromosomes excluded. Samples and features with more than 30% missing rate in miRNA expression, mRNA expression, and promoter CpG methylation data were removed, and the remaining missing values were imputed using the K-nearest neighbor (KNN) method [39]. The miRNA and mRNA expression then were ${\log}_2\left(x+1\right)$ transformed to mitigate the impact of extreme values or outliers in the data. We further removed mRNAs and promoter CpG methylations with low variation across samples. Specifically, we removed features from mRNA data that were below the 15th percentile concerning mean, variance, and coefficient of variation and features from methylation data that were below the 25th percentile concerning mean, variance, and coefficient of variation. After preprocessing, we retained 10,374 mRNAs, 388 miRNAs, and 20,681 promoter CpG methylation features for the 285 KIRC patients, and 10,154 mRNAs, 437 miRNAs, and 20,680 promoter CpG methylation features for the 206 KIRP patients.

#### Evaluation of the subtyping results on real data applications

We utilized multi-omics data from two prominent types of renal cell carcinoma, namely KIRC and KIRP, to assess the clustering performance of Meta-SNF and carried out a series of downstream analyses. First, we compared Meta-SNF with SNF, intNMF, and ConsensusClusterPlus in subtyping KIRC and KIRP patients. We identified the optimal number of clusters using the ‘eigengap’ metric [31]. After obtaining the label information from the four methods, we performed the log-rank test to evaluate the difference in survival outcomes between subtypes. Then we assessed the clustering performance under different subtyping results using the clustering evaluation metrics such as the Dunn index [40], Calinski Harabasz Score index [41], and Davies-Bouldin index [42]. Due to fundamental differences in the matrices used for clustering by intNMF and ConsensusClusterPlus (consensus matrices) and those employed by Meta-SNF and SNF (similarity matrices), a direct comparison among the three metrics is not feasible. Therefore, we exclusively focused on the comparison between Meta-SNF and SNF. We also constructed a *Cox* proportional hazards model incorporating clinical covariates to assess the risk levels between different subtypes and identify factors that may influence patient survival outcomes.

To uncover prognostic differences among subtypes at the molecular level, we performed a series of bioinformatics analyses. We applied the weighted gene co-expression network analysis (WGCNA) method on the mRNA expression data to find gene modules associated with the prognosis of two cancers using the R package WGCNA [43]. We then performed Gene Ontology (GO) [44] and Kyoto Encyclopedia of Genes and Genomes (KEGG) [45] analysis on the genes in the key modules using the online tool KOBAS 3.0 [46]. Additionally, we refined our selection of hub genes within these key gene modules, which were then subjected to drug sensitivity and expression correlation analysis based on the Genomics of Drug Sensitivity in Cancer (GDSC) [47] and the Cancer Therapeutics Response Portal (CTRP) [48] databases. Finally, we employed the R package PROGENy [49] to identify variations in 14 signaling pathways based on gene expression data across subtypes.

## Results

### Simulation results

**Figure 2:**
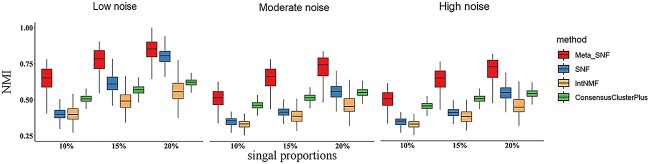
Distribution of NMI values under different signal-to-noise levels in 1000 simulation runs.

Our simulation study revealed that Meta-SNF outperformed the original SNF method, intNMF, and ConsensusClusterPlus in subtyping accuracy across all signal-to-noise settings, especially in the presence of higher noise levels. We consolidated the NMI values from different subtyping methods into Table 1 and depicted their performance distribution across 1000 simulations in Fig. 2 using boxplots. The analysis revealed that the clustering efficacy of all methods improves with increased signal proportions and diminishes with higher noise levels. From the data presented in Table 1 and Fig. 2, it is evident that Meta-SNF significantly enhances the accuracy of sample label identification when compared to SNF. For example, under conditions of 20% signal strength and high noise levels (${\sigma}^2=12$), Meta-SNF achieved an NMI value of 0.665, surpassing SNF’s value of 0.43, intNMF’s value of 0.387 and ConsensusClusterPlus’ value of 0.514. This demonstrates the robust performance of Meta-SNF for data integration under high noise conditions.

**Table 1:** The clustering performance of Meta-SNF and other methods measured by NMI

**Method**	**SimData1 (** ${\boldsymbol{\sigma}}^{\mathbf{2}}=\mathbf{6}$ **)**				**SimData2 (** ${\boldsymbol{\sigma}}^{\mathbf{2}}=\mathbf{9}$ **)**				**SimData3 (** ${\boldsymbol{\sigma}}^{\mathbf{2}}=\mathbf{12}$ **)**		
	**10% signal**	**15% signal**	**20% signal**		**10% signal**	**15% signal**	**20% signal**		**10% signal**	**15% signal**	**20% signal**
Meta-SNF	**0.670** **(0.095)**	**0.783** **(0.090)**	**0.839** **(0.085)**		**0.532** **(0.080)**	**0.674** **(0.091)**	**0.756** **(0.091)**		**0.437** **(0.068)**	**0.570** **(0.082)**	**0.665** **(0.093)**
SNF	0.401 (0.040)	0.612 (0.064)	0.798 (0.058)		0.348 (0.031)	0.420 (0.044)	0.559 (0.063)		0.307 (0.034)	0.366 (0.029)	0.430 (0.039)
intNMF	0.402 (0.051)	0.494 (0.063)	0.563 (0.078)		0.331 (0.031)	0.391 (0.053)	0.461 (0.063)		0.309 (0.032)	0.337 (0.035)	0.387 (0.048)
Consensus- ClusterPlus	0.508 (0.026)	0.569 (0.032)	0.620 (0.027)		0.462 (0.028)	0.514 (0.028)	0.551 (0.030)		0.411 (0.031)	0.477 (0.026)	0.514 (0.028)

Note: The values of NMI are presented as the mean (standard deviation) of 1000 simulation runs. The one with the best performance is highlighted with bold fonts.

### Overall subtyping performance of KIRC and KIRP

We compared Meta-SNF with other methods for subtyping KIRC and KIRP patients, namely SNF, intNMF, and ConsensusClusterPlus. All the optimal cluster numbers were determined using the eigengap metric. In both datasets, Meta-SNF and SNF respectively identified four and three subtypes while the subtypes identified by the other two methods were not consistent (see Table 2). The statistics of Log-rank test among the identified subtypes showed that Meta-SNF had better performance with the smallest *p-value*. This indicates that the four subtypes identified by Meta-SNF show greater distinction in survival rate relative to the other three methods.

**Table 2:** Comparison of subtyping results of different integration methods

**Cancer types**	**Item**	**Meta-SNF**	**SNF**	**ConsensusClusterPlus**	**intNMF**
KIRC	Number of clusters	4	3	3	4
	log rank *P*-value	**4.05E-09**	3.62E-05	3.2E-04	6.58E-05
KIRP	Number of clusters	4	3	4	3
	log rank *P*-value	**8.43E-06**	2.54E-04	0.0003	2.9E-04

Note: ‘Number of clusters’ relates to the optimal number of clusters based on the eigengap metric; ‘log rank P-value’ is the P-value obtained by the log rank test.

**Figure 3:**
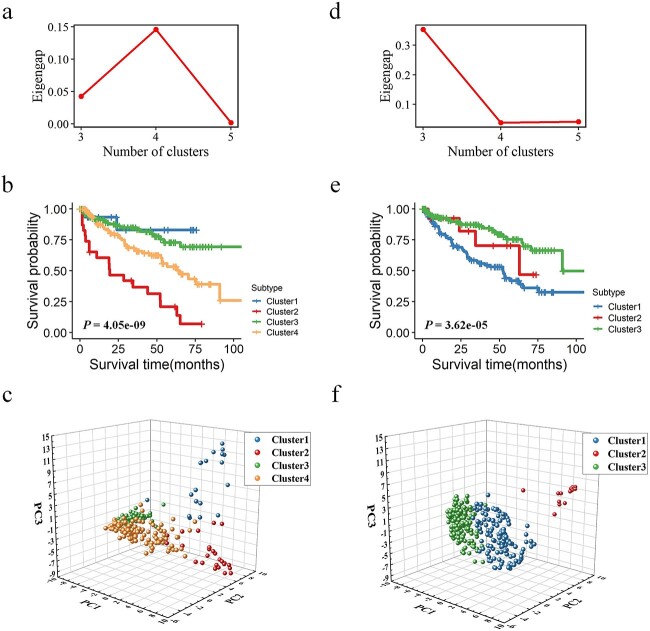
Subtyping results of KIRC. (a) Plot of eigengap (y-axis), showing four as the optimal number of clusters for Meta-SNF. (b) Kaplan–Meier curves of the four subtypes identified by Meta-SNF. (c) 3D scatter plot of the first three principal components based on the fused similarity matrix with Meta-SNF. (d, e, and f) show the corresponding results of SNF.

**Figure 4:**
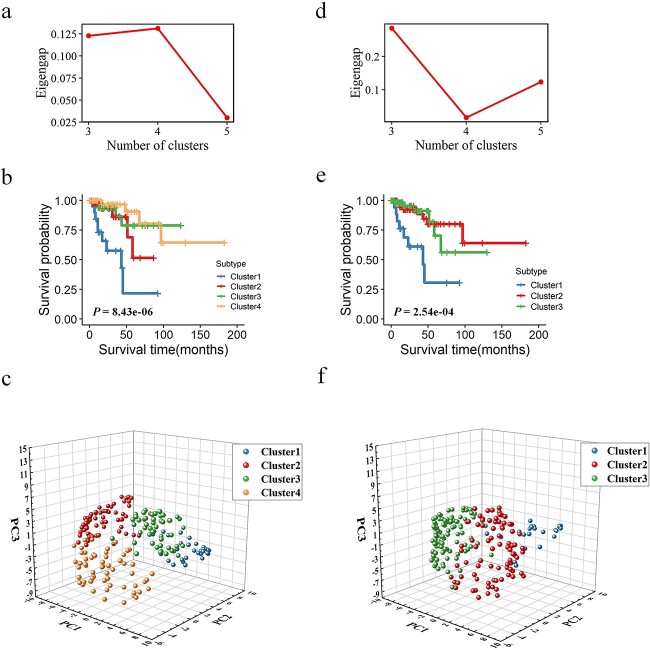
Subtyping results of KIRP. (a) Plot of eigengap (y-axis), showing four as the optimal number of clusters for Meta-SNF. (b) Kaplan–Meier curves of the four subtypes identified by Meta-SNF. (c) 3D scatter plot of the first three principal components based on the fused similarity matrix with Meta-SNF. (d, e, and f) show the corresponding results of SNF.

Next, building on previous research [50–52], we assessed the clustering efficacy, focusing primarily on Meta-SNF and SNF, across scenarios where the number of clusters ranged from 3 to 5, employing three distinct clustering metrics for evaluation. As shown in Table 3, Meta-SNF outperformed SNF across most clustering results, which indicates that the subtypes from Meta-SNF are more distinct and well-separated. We drew the plot of eigengap for different clusters in KIRC and KIRP to determine the optimal cluster number (See Fig. 3a and Fig. 4a). Patients of KIRC were optimally divided into four subtypes by using Meta-SNF and 3 subtypes by SNF, and patients of KIRP were optimally divided into four subtypes by Meta-SNF and 3 subtypes by SNF. We can observe that the Kaplan–Meier curve drawn based on the subtypes from Meta-SNF demonstrates a stronger separation between subtypes compared to SNF (also revealed by the log-rank test), which reveals a more significant prognosis difference (Fig. 3b and Fig. 4b). Furthermore, we draw 3D scatter plots using the top three principal components (PCs) based on the fusion networks to check if their optimal subtyping results can be distinguished. Figure 3c and Fig. 4c summarize pictorially that the distribution of components from Meta-SNF in the same cluster is more concentrated, while it is more scattered in different clusters.

**Table 3:** The clustering performance of Meta-SNF and SNF on KIRC and KIRP

**Number of clusters**	**Method**	**KIRC**			**KIRP**		
		**DBI** ^**a**^	**CH** ^**b**^	**DI** ^**c**^	**DBI** ^**a**^	**CH** ^**b**^	**DI** ^**c**^
3	Meta-SNF	**3.424**	**13.444**	**0.086**	**2.595**	**20.136**	**0.118**
	SNF	3.475	15.767	0.018	2.749	17.942	0.079
4	Meta-SNF	**3.509**	**12.496**	**0.036**	**2.575**	**18.823**	**0.060**
	SNF	3.912	12.382	0.019	3.011	14.842	0.057
5	Meta-SNF	**3.515**	**11.413**	**0.023**	**2.544**	**16.429**	**0.035**
	SNF	3.624	10.449	0.013	2.913	13.239	0.044

^a^DBI stands for ‘Davies-Bouldin Index’. A lower DBI value indicates better clustering.

^b^CH stands for “Calinski-Harabasz Index. A higher CH value indicates better clustering.

^c^DI stands for ‘Dunn index’. A higher DI indicates better clustering.

^d^The one(s) with the better performance is(are) highlighted in bold fonts.

#### Analysis of KIRC subtypes identified by Meta-SNF

The 285 KIRC patients were divided into four subtypes (19 patients in Cluster 1; 25 patients in Cluster 2; 128 patients in Cluster 3; 113 patients in Cluster 4) with significant prognosis differences (see Fig. 3b, ${\chi}^2$=42, log-rank *p-value* = 4.05E-09) using Meta-SNF. We summarized the relevant clinical data for each cluster in Table 4, in which Cluster 2 and Cluster 4 exhibited a poorer prognosis and simultaneously showed a higher proportion of patients with advanced pathology. Patients in the two clusters will be the primary focus of subsequent analysis.

**Table 4:** Baseline clinical data for different subtypes of KIRC

**Items**	**Cluster 1**	**Cluster 2**	**Cluster 3**	**Cluster 4**
Number of patients (*n*, %)	19(6.67)	25(8.77)	128(44.91)	113(39.65)
Age at initial diagnosis (year, *mean ± sd*)	58.84 ± 9.23	61.00 ± 11.15	60.84 ± 10.59	58.98 ± 10.69
Gender (*n*, %)				
Male	11(57.89)	18(72)	75(58.60)	81(71.68)
Female	8(42.11)	7(28)	53(41.40)	32(28.31)
Vital status (*n,* %)				
Survival	17(89.47)	6(24)	107(83.59)	73(64.60)
Death	2(10.53)	19(76)	21(16.41)	40(35.40)
Pathologic stage (*n, %*)				
I	12(63.16)	6(24)	69(53.91)	51(45.13)
II	3(15.79)	1(4)	14(10.94)	11(9.74)
III	3(15.79)	7(28)	25(19.53)	29(25.66)
IV	1(5.26)	11(44)	20(15.62)	22(19.47)

We performed multivariable *Cox* regression analysis to assess the associations between subtypes and survival outcomes while adjusting for the effects of clinical factors such as age at initial diagnosis, gender, and pathological stage. The result in Table 5 shows that patients in Cluster 2 and Cluster 4 have 4.043-fold and 1.819-fold higher risk of death than those in Cluster 3, respectively.

**Table 5:** Cox regression analysis of 285 KIRC patients

**Variables**	***b* (*S.E*)**	***Z***	***P***	***HR* (95% *CI*)**
Subtypes				
Cluster 1	0.133(0.750)	0.178	0.859	1.142(0.263-4.969)
Cluster 2^*^	1.397(0.330)	4.225	**2.39E-05**	4.043(2.115-7.730)
Cluster 4^*^	0.598(0.272)	2.199	**0.028**	1.819(1.067-3.102)
Age at initial diagnosis	0.020(0.012)	1.638	0.101	1.020(0.996-1.045)
Gender	−0.219(0.243)	−0.902	0.367	0.803(0.499-1.293)
Pathologic stage				
II	0.444	0.840	0.401	1.559(0.553-4.390)
III^*^	1.194	3.363	**7.72E-04**	3.299(1.645-6.616)
IV^*^	2.070	6.258	**3.90E-10**	7.923(4.413-15.150)

Note: ^*^Shows statistically significant (*P* < 0.05); Here we take cluster 3 with better prognosis as the reference group for comparison and take Pathologic stage I as the reference stage for comparison.

#### Analysis of KIRP subtypes identified by Meta-SNF

The 206 KIRP patients were divided into four subtypes (30 patients in Cluster 1; 67 patients in Cluster 2; 49 patients in Cluster 3; and 60 patients in Cluster 4) with significant differences in overall survival (see Fig. 4b, ${\boldsymbol{\chi}}^{\mathbf{2}}$=26.3, log-rank *p-value* = 8.43E-06) by Meta-SNF. The relevant clinical data for each cluster was summarized in Table 6. In our subsequent analysis, we primarily focused on patients in Cluster 1 and Cluster 2 with poor prognosis.

**Table 6:** Baseline clinical data for different subtypes of KIRP

**Items**	**Cluster 1**	**Cluster 2**	**Cluster 3**	**Cluster 4**
Number of patients (*n*, %)	30(14.56)	67(32.52)	49(23.79)	60(29.13)
Age at initial diagnosis (year, mean ± sd)	53.97 ± 13.84	60.78 ± 10.02	64.37 ± 9.54	61.23 ± 10.82
Gender (*n*, %)				
Male	11(36.67)	53(79.10)	31(63.27)	51(0.85)
Female	19(63.33)	14(20.90)	18(36.73)	9(0.15)
Vital status (*n*, %)				
Survival	21(0.7)	62(92.54)	45(91.84)	56(93.33)
Death	9(0.3)	5(7.46)	4(8.16)	4(6.67)
Pathologic stage (*n, %*)				
I	12(0.4)	57(85.07)	26(53.06)	42(70.00)
II	1(3.33)	3(4.48)	1(2.04)	9(15.00)
III	11(36.67)	5(7.46)	19(38.78)	8(13.33)
IV	6(0.2)	2(2.99)	3(6.12)	1(1.67)

Similarly, we fitted a multivariable *Cox* proportional hazards model to assess the variations in prognosis among different subtypes. The result was summarized in Table 7, among which patients in Cluster 1 and Cluster 2 had 8.677-fold and 4.674-fold higher risk of death than those in Cluster 4, respectively.

**Table 7:** Cox regression analysis of 206 KIRP patients

**Variables**	***b* (*S.E*)**	***Z***	***P***	***HR* (95% *CI*)**
Subtypes				
Cluster 1^*^	2.161(0.740)	2.919	**0.004**	8.677(2.034-37.025)
Cluster 2^*^	1.536(0.772)	1.990	**0.047**	4.647(1.024-21.097)
Cluster 3	0.429(0.816)	0.526	0.599	1.535(0.310-7.595)
Age at initial diagnosis	0.011(0.024)	0.448	0.654	1.011(0.965-1.058)
Gender	0.252(0.555)	0.455	0.649	1.287(0.434-3.820)
Pathologic stage				
II	0.319(1.097)	0.291	0.771	1.376(0.160-11.814)
III	0.929(0.654)	1.421	0.155	2.533(0.703-9.124)
IV^*^	2.417(0.652)	3.709	**2.08E-04**	11.216(3.127-40.234)

Note: ^*^Shows statistically significant (*P* < 0.05); Here we take Cluster 4 with better prognosis as the reference group for comparison and take Pathologic stage I as the reference stage for comparison.

#### Co-expression network construction and core gene modules identification of KIRC

**Figure 5:**
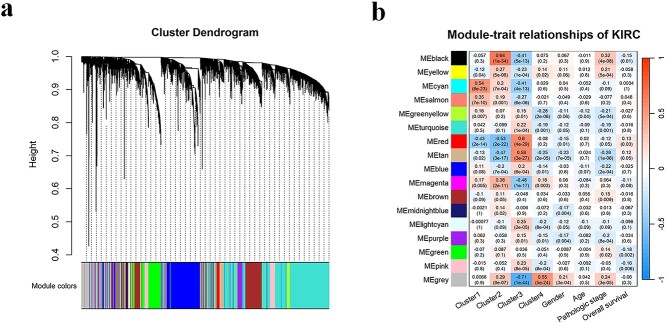
WGCNA analysis of the KIRC data. (a) Dendrogram representing hierarchical clustering of identified co-expressed modules in KIRC mRNA expression data. (b) Heatmap visualizing the correlation between eigengene of modules and clinical traits of KIRC. Each row represents a color module, and each column represents a clinical feature. Each cell is filled with the correlation and *p-value*.

To identify gene modules that may influence the progression of KIRC patients, we performed WGCNA analysis on the mRNA expression data. The optimal soft-thresholding power was set at 7 based on the criterion of scale-free fit topology index (*R^2^* = 0.856). We transformed the adjacency matrix into a topological overlap matrix and employed a dynamic tree-cutting algorithm to detect gene modules, and further consolidated the related modules using a height cutoff of 0.25. Subsequently, we identified 16 co-expression gene modules (see Fig. 5a). We further calculated the correlation between module genes and features of our interest such as subtypes (Clusters 1–4) and clinical traits (age at initial diagnosis, gender, pathologic stage and overall survival), and visualized their associations using heatmap. As shown in Fig. 5b, both the black module and the red module show a strong correlation with the poor prognosis groups, Cluster 2 and Cluster 3. Specifically, the black module has a correlation coefficient of 0.64 (*p-value* = 1E-34) with Cluster 2, and shows moderate association with the pathological stage (*r* = 0.32, *p-value* = 4E-08). The red module has a correlation coefficient of 0.6 (*p-value* = 4E-29) with Cluster 3 and shows moderate associations with Cluster 1 (*r* = −0.43, *p-value* = 2E-14) and Cluster 2 (*r* = −0.53, *p-value* = 2E-22). We regarded the black and red modules as key modules of KIRC and selected a total of 1076 genes in the two modules for further bioinformatics analysis.

#### Co-expression network construction and core gene modules identification of KIRP

**Figure 6:**
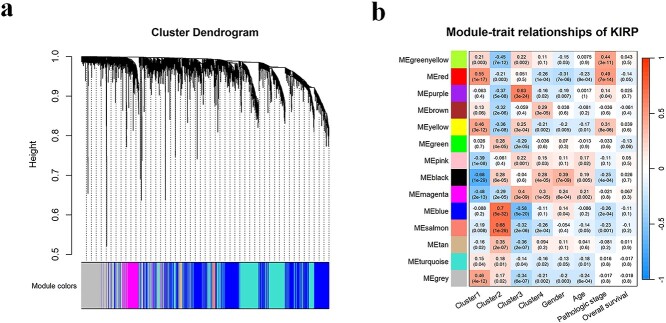
WGCNA analysis of the KIRP data. (a) Dendrogram representing hierarchical clustering of identified co-expressed modules in KIRP mRNA expression data. (b) Heatmap visualizing the correlation between eigengene of modules and clinical traits of KIRP. Each row represents a color module, and each column represents a clinical feature. Each cell is filled with the correlation and *p-value*.

The WGCNA was also performed on the mRNA expression data of KIRP patients to find gene modules correlated with prognosis. According to the scale-free topology index (*R^2^* = 0.827), we set the optimal soft-thresholding power at 7. After converting the adjacency matrix into a topological overlap matrix, we identified 13 gene modules (see Fig. 6a) following the same methodology as in Section (3.2.3). We further calculated the correlation between the 13 modules and factors such as subtypes (Clusters 1–4) and clinical traits (age at initial diagnosis, gender, pathologic stage and overall survival), and visualized their associations with heatmap (see Fig. 6b). We noticed that among all 13 modules, both the red and the blue module have a close correlation with the traits that we focused on. Explicitly, the red module has a positive correlation coefficient of 0.55 (*p-value* = 1E-17) with Cluster 1 which has a worse prognosis and shows moderate associations with the pathological stage (*r* = 0.44, *p-value* = 3E-11). The blue module has a correlation coefficient of 0.7 (*p-value* = 5E-32) with Cluster 2 and shows a negative correlation with Cluster 3 (*r* = −0.58, *p-value* = 5E-20). We regarded the red and the blue modules as key modules of KIRP and selected a total of 2082 genes in the two modules for subsequent bioinformatics analysis.

#### Enrichment analysis of genes in the key modules for KIRC and KIRP

We performed GO and KEGG analyses of genes in the key modules of KIRC and KIRP respectively, to extract meaningful biological information and evaluate functional relevance.

**Figure 7:**
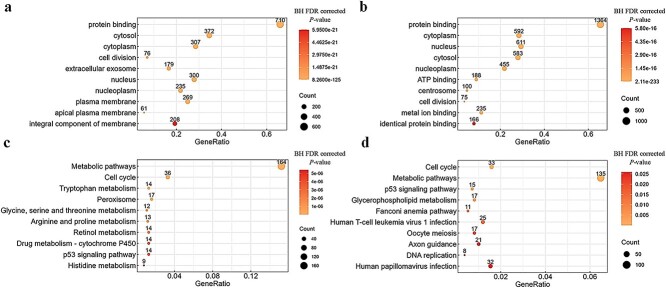
Enrichment analysis of genes in the key modules for KIRC and KIRP. (a) The top 10 enriched GO terms of genes in KIRC. (b) The top 10 enriched GO terms of genes in KIRP. (c) The top 10 enriched KEGG pathways of genes in KIRC. (d) The top 10 enriched KEGG pathways of genes in KIRP.

For the GO analysis, we identified 597 biological function categories (BH FDR corrected *p-value* < 0.05) in KIRC and 372 biological function categories (BH FDR corrected *p-value* < 0.05) in KIRP. The ten most statistically significant terms in KIRC include ‘protein binding’, ‘cytosol’, ‘cytoplasm’, etc. (see Fig. 7a), and the ten most statistically significant terms in KIRP include ‘protein binding’, ‘cytoplasm’, ‘nucleus’, etc. (see Fig. 7b).

The enrichment patterns of the key module genes in two types of cancers were remarkably similar, with six out of the top ten biological terms being identical. Among these, 710 genes in KIRC and 1364 genes in KIRP are enriched in the GO term of ‘protein binding’ with a high gene ratio. Functional changes associated with this GO term may play a central role in RCC development.

For the KEGG analysis, genes in the key modules of KIRC were enriched in 87 pathways (BH FDR corrected *p-value* < 0.05), whereas genes in the key modules of KIRP were enriched in 16 pathways (BH FDR corrected *p-value* < 0.05). Figure 7c and d respectively show the top 10 pathways with *p-values* for the KIRC and KIRP data. Similarly, in the KEGG analysis, key genes of both KIRC and KIRP exhibited enrichment in the Metabolic pathways, Cell cycle, and p53 signaling pathway. Further investigation into the genes associated with these terms and pathways, as well as their functional roles in KIRC and KIRP, could provide valuable insights regarding disease mechanisms and potential therapeutic targets.

#### Hub gene identification and drug sensitivity detection

**Figure 8:**
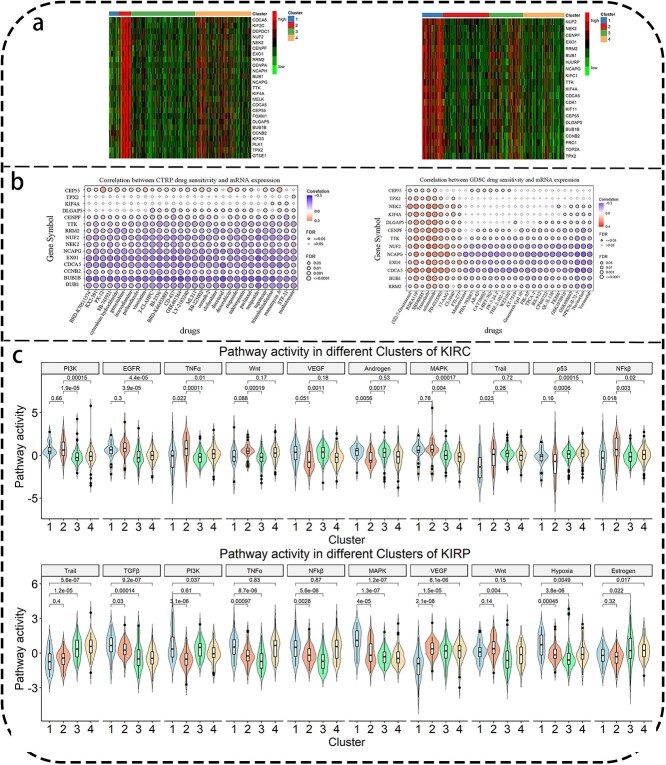
(a) Expression heatmap of candidate genes. The expression of the 25 hub candidate genes across four clusters of KIRC (left). The expression of the 21 hub candidate genes across four clusters of KIRP (right). The four colored bars at the top represent subtypes 1–4 from left to right. (b) Bubble chart showing correlation between mRNA expression and drug sensitivity based on CTRP (left) and GDSC (right). The x-axis represents different drugs, while the y-axis represents genes. The size of the circles indicates the value of FDR *p-value*, and different colors represent the degree of correlation. (c) The violin plot of the top 10 pathway activities across different subtypes identified by Meta-SNF in KIRC and KIRP.

To identify hub genes influencing the progression of RCC, we conducted screenings for genes in key modules of KIRC and KIRP. Candidate genes were defined as those exhibiting strong correlations with module eigengenes (ModuleMembership correlation >0.9) and clinical traits (TraitSignificance correlation >0.3). Under the filtering criteria, we screened 25 candidate genes from the red and black module in KIRC, and 21 candidate genes from the red and blue module in KIRP. As shown in Fig. 8a, the candidate genes in KIRC and KIRP display varying expression levels across different subtypes. Specifically, the expression of candidate genes in clusters with the worst prognosis was higher than others. We ultimately considered the intersection of the two sets of candidate genes as hub genes of RCC for further investigation. The intersection list consists of a total of 15 genes: *BUB1*, *BUB1B*, *CCNB2*, *CDCA5*, *CENPF*, *CEP55*, *DLGAP5*, *EXO1*, *KIF4A*, *NCAPG*, *NEK2*, *NUF2*, *RRM2*, *TPX2*, and *TTK*.

Drug sensitivity analysis was conducted on the 15 hub genes using the Gene Set Cancer Analysis platform (GSCA, http://bioinfo.life.hust.edu.cn/GSCA) [53], which integrates drug sensitivity data and mRNA expression data from both the GDSC [47] and CTRP [48] databases. GCSA performs sensitivity analysis by calculating correlations between drug IC50 values and genes, along with corresponding FDR-adjusted *p-values*. Specifically, drug-gene pairs with an absolute correlation coefficient > 0.1 and FDR-adjusted *p-value* < 0.05 were retained. A score for each gene-drug pair was calculated via multiplying the FDR-adjusted *p-value* with-log10 transformation and the absolute value of the correlation coefficient. Subsequently, final scores were computed for each drug, and the 30 highest ranked drugs were visualized in a bubble plot. Figure 8b presented the correlation between hub gene expression and sensitivity for the top 30 drugs in both the CTRP and GDSC datasets across pan-cancer data. The positive correlation between genes and drugs indicates that higher gene expression may lead to drug resistance, while negative correlation suggests that higher gene expression may make drug sensitive, thereby enhancing its effectiveness in combating diseases or symptoms.

#### Pathway activity analysis among the identified molecular subtypes by Meta-SNF

We estimated the activities of 14 critical biological signaling pathways across the different subgroups of KIRC and KIRP patients. Kruskal–Walli test was used to identify biological pathways and the threshold was set as FDR-adjusted *p-value*$<\mathbf{0.05}$. We identified 12 and 13 pathways showing significant differences among KIRC and KIRP subtypes, respectively. The top 10 pathways for both KIRC and KIRP are illustrated in Fig. 8c. The poor prognoses observed in Cluster 2 of KIRC and Cluster 1 of KIRP may be linked to the activity levels of these pathways.

#### Robustness and adaptability of Meta-SNF to other cancer subtyping

We also applied Meta-SNF to two other datasets of Low-Grade Gliomas (LGGs) from TCGA [28] and Chinese Glioma Genome Atlas (CGGA) [54] to check the robustness and adaptability of Meta-SNF in cancer subtyping. We considered three omics data types of each cancer, namely miRNA expression, mRNA expression, gene-level DNA methylation data from CGGA and promoter CpG methylation from TCGA.

We employed the same data preprocessing procedure as KIRC and KIRP. Finally, we had 11,923 mRNAs, 827 miRNAs, and 6107 gene-level DNA methylations for the 86 LGG patients from CGGA, and 10,461 mRNAs, 501 miRNAs, and 20,681 promoter CpG methylation features for the 499 LGG patients from TCGA.

LGG patients from both CGGA and TCGA were classified into four subtypes with significant prognostic differences using Meta-SNF (see Fig. S1 in the Supplementary Materials). The log-rank test showed significant difference in survival rates among the four subtypes in the CGGA data (*p-value* = 1.77E-07) and in the TCGA data (*p-value* < 2E-16), indicating the robustness and adaptability of Meta-SNF for cancer subtyping across different datasets.

## Discussion

RCC exhibits a high degree of heterogeneity, coupled with elevated rates of recurrence and metastatic risk. Integrating multi-omics data is of significant importance to understand the molecular mechanisms and drive the development of treatment strategies for RCC. In recent years, the surge in multi-omics data has driven the creation of various integration strategies, each with their unique strengths and limitations. This study delves into harnessing the synergistic potential of similarity-based and intermediate integration techniques to forge a more effective multi-omics data integration method termed Meta-SNF by integrating the NMF algorithm with the SNF process. Through simulation studies, we validated the effectiveness and superiority of Meta-SNF over its counterparts. It demonstrated enhanced capability in handling data noise and achieved high-precision subtype identification relative to its predecessors SNF, intNMF, and ConsensusClusterPlus, across diverse signal-to-noise ratios.

In an application to two RCC cancer types, both KIRC and KIRP were classified into four subtypes by Meta-SNF with significant differences in survival outcomes. We focused on the subtypes with poor prognosis, firstly conducting weighted co-expression analysis to identify gene modules and hub genes that may explain these outcomes. In the enrichment analysis, we observed that the pathway enrichment and biological term enrichment patterns of the key genes in KIRC and KIRP are similar. Among those shared pathways, the Metabolic pathway has shown to be an important targets for RCC-therapies and crucial areas for future research [55]. The Cell cycle governs the rate of cell division and proliferation [56], and the breakdown of cell cycle regulation is believed to be the initial stage in carcinogenesis, playing a significant role in tumor invasion and the development of metastases [57]. Liu et al. demonstrated that the concurrent activation of p53 and HIF1α, achieved through the use of antagonists, effectively induces apoptosis in RCC cells [58]. This suggests that a therapeutic strategy combining murine double minute 2 (MDM2) antagonists with mechanistic target of rapamycin (mTOR) inhibitors could be highly effective in treating RCC. Among the 15 identified hub genes, the overexpression of *BUB1B* has been demonstrated to be correlated with elevated activity in the p53 signaling pathway [59]. Knockdown experiments reducing *BUB1B* through knockdown experiments has been found to inhibit cell growth and invasion of RCC [60]. Altered expression levels of never in mitosis gene-A-related kinase 2 (*NEK2*) can impact drug resistance and tumor progression [61]. In vitro studies by Feng et al. [62] show that *NEK2* overexpression promotes the proliferation, migration, and invasion of RCC cells. Besides, the majority of other identified hub genes may be promising potential biomarkers of prognosis in RCC or other cancers, as has been illustrated in the literature [63–72].

Drug sensitivity analysis based on hub genes provides potential treatment options for RCC therapy. Among the top 30 ranked drugs in CTRP and GDSC, *gemcitabine* [73], *vincristine* [74], *docetaxel* [75], *paclitaxel* [76], *methotrexate* [77], and *vorinostat* [78] have been widely used in the treatment of RCC patients, the other drugs may be potential options for RCC treatment. The onset and progression of cancer are often associated with abnormal biological pathway signaling activity. In pathway activity analysis, we identified several statistically different biological pathways between subtypes in both KIRC and KIRP. Among them, increased Androgen receptor expression has been considered as a favorable prognostic indicator in cancer patients [79] and the poor prognosis of Cluster 2 in KIRC may be related to its lower expression. The activity of the tumor necrosis factor-related apoptosis-inducing ligand (TRAIL) and vascular endothelial growth factor (VEGF) pathways in Cluster 1 of KIRP patients is lower than in other groups. Prior studies have revealed that TRAIL can selectively eliminate tumor cells with minimal effects on normal cells [80]. Concurrently, the VEGF pathway plays a significant role in angiogenesis, therapy resistance, and metastasis, particularly in the development of renal carcinoma. VEGF-targeting agents have demonstrated robust efficacy at standalone treatments in metastatic RCC [81, 82], underscoring their therapeutic potential. These findings further confirmed the advantages of Meta-SNF in molecular subtyping by offering valuable biological insights and providing underlying targets for therapies in clinical decision-making.

The transformation of thousands of genes along with surrounding noise into a handful of metagenes enables more accurate calculation of patient similarity and reduces the risk of detecting potentially spurious associations during clustering. Nonetheless, several limitations should be acknowledged in our study. Suitable initialization procedures can significantly increase the iteration speed of the NMF algorithm and reduce the overall error between the original data and the metagene matrix [83]. In this study, we utilized the original random strategy to generate the initial $W$ and $H$. This makes the construction of the metagene matrix susceptible to the number of iterations, which may contribute to larger variance in the simulation results of Meta-SNF compared to other methods. It is well recognized that incorporating prior biological information into the process of multi-omics data integration can improve subtype identification. This insight has led us to consider that associating metagenes with biological factors, such as pathological staging, survival outcomes, or prognostic performance, may yield more biologically meaningful subtyping results in future studies. Currently, the validation process of Meta-SNF primarily relies on the simulation study and the application of two types of RCC. Conducting a comprehensive analysis across a wider array of other multi-omics datasets would offer enhanced insights into Meta-SNF’s capability in tumor subtype identification. Moreover, the biomarkers we identified should be interpreted with caution since the causality between them and RCC remain unclear and require further biological validation. Recognizing these limitations, we plan to explore these avenues in our future research endeavors, aiming to further validate and refine our work.

In the additional analysis of the two LGG datasets, Meta-SNF demonstrated its robust ability to identify subtypes with significant prognostic differences. In conclusion, our proposed Meta-SNF is an innovative strategy for integrating multi-omics data, achieving higher accuracy of subtype identification than the original SNF method and other state-of-the-art methods. It effectively captures biological signals and complementary insights from a range of omics data types. Its application to RCC datasets provided diverse biological insights that are highly valuable for informing clinical decision-making processes in the treatment of RCC. Beyond integrating multi-omics of bulk data, it can also be adapted for unsupervised subtyping studies of single-cell multi-omics data and other multimodal datasets.

**Key Points**

- Our research focused on subtype identification of renal cell carcinoma with multi-omics data. We proposed a novel multi-omics integration method called Meta-SNF for RCC subtype identification.
- Simulation study revealed that Meta-SNF outperformed the original SNF method as well as other state-of-the-art methods under different settings.
- Application to two RCC datasets identified meaningful subtypes and key cancer-associated biomarkers.
- Meta-SNF has potential broad applications for unsupervised subtyping studies of multimodal datasets.

## Supplementary Material

Meta-SNF-supplementary_materials-R1_bbae606

## Data sharing statement

The RCC data and source code of Meta-SNF used for this study are available at https://github.com/CongcongJia/Meta-SNF.git.
