# Supplementary material for: A metagene based similarity network fusion approach for multi-omics data integration identified novel subtypes in renal cell carcinoma
Source: Brief Bioinform. 2024 Nov 19;25(6):bbae606. doi: 10.1093/bib/bbae606 (PMC11576078; doi:10.1093/bib/bbae606)
Supplement: Meta-SNF-supplementary_materials-R1_bbae606 [file meta-snf-supplementary_materials-r1_bbae606.docx]

**Supplemental Material for^[[1]](#footnote-1)^ “A metagene based similarity network fusion approach for** **multi-omics data integration identified novel subtypes in renal cell carcinoma”**

Congcong Jia^a,b,1^, Tong Wang^c,a,1^, Dingtong Cui^a, b^, Yaxin Tian^c,a^, Gaiqin Liu^a, b^, Zhaoyang Xu^c,a^, Yanhong Luo^a, b^, Ruiling Fang^a,b^, Hongmei Yu^a,b^, Yanbo Zhang^a,b^, Yuehua Cui^d,*^, Hongyan Cao^a,b,*^

^a^*Department of Health Statistics, Shanxi Provincial Key Laboratory of Major Diseases Risk Assessment, School of Public Health, Shanxi Medical University, Taiyuan, Shanxi 030001, PR China*

^b^*MOE Key Laboratory of Coal Environmental Pathogenicity and Prevention, Shanxi Medical University, 030001 Taiyuan, Shanxi, China.*

^c^*Academy of Medical Sciences, Shanxi Medical University, Taiyuan, Shanxi 030001, PR China*

^d^*Department of Statistics and Probability, Michigan State University, East Lansing, MI 48824, USA*

**Supplementary Figure**


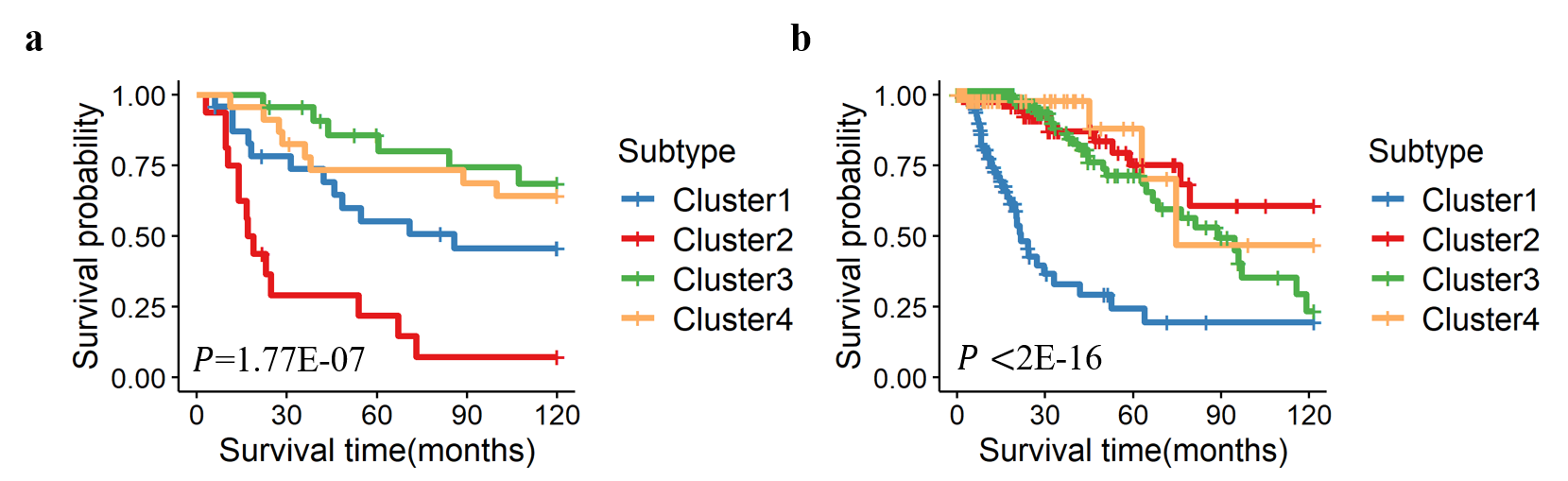


**Figure S1.** Subtyping results of 86 LGG patients from CGGA(a) and 499 LGG patients from TCGA(b) using Meta-SNF.

1. *Corresponding author: [cuiy@msu.edu](mailto:cuiy@msu.edu) (Y. Cui); caohy@sxmu.edu.cn (H. Cao)

   ^1^Contributed equally as first authors. [↑](#footnote-ref-1)
